# Development and validation of a faecal immunochemical test-based model in the work-up of patients with iron deficiency anaemia

**DOI:** 10.3389/fmed.2024.1407812

**Published:** 2024-06-25

**Authors:** Goretti Hernández, Enrique Quintero, Dalia Morales-Arraez, Guillermo García Rayado, Gonzalo Hijos-Mallada, Nereida Fernández-Fernández, Luisa de Castro-Parga, María Victoria Álvarez-Sánchez, Carolina Olano, Daniel Rodríguez-Alcalde, Carla Amaral-González, Inmaculada Alonso-Abreu, David Nicolás-Pérez, Marta Carrillo-Palau, Enrique González-Dávila, Antonio Z. Gimeno-García

**Affiliations:** ^1^Servicio de Aparato Digestivo, Hospital Universitario de Canarias, Santa Cruz de Tenerife, Spain; ^2^Departamento de Medicina Interna, Instituto Universitario de Tecnologías Biomédicas (ITB) & Centro de Investigación Biomédica de Canarias (CIBICAN), Universidad de La Laguna, San Cristóbal de La Laguna, Spain; ^3^Hospital Clínico Universitario Lozano Blesa, Zaragoza, Spain; ^4^Hospital Álvaro Cunqueiro de Vigo, Pontevedra, Spain; ^5^Complejo Hospitalario Universitario de Vigo, Vigo, Spain; ^6^Complejo Hospitalario Universitario de Pontevedra, Pontevedra, Spain; ^7^Clínica de Gastroenterología. Facultad de Medicina, Montevideo, Uruguay; ^8^Hospital Universitario de Móstoles, Madrid, Spain; ^9^Departamento de Matemáticas, Estadística e Investigación Operativa, Instituto IMAULL, Universidad de La Laguna, San Cristóbal de La Laguna, Spain

**Keywords:** colorectal cancer, cancer-diagnosis, colonoscopy, diagnostic tests, endoscopy, iron deficiency, anaemia

## Abstract

**Objective:**

In patients with iron deficiency anaemia (IDA), the diagnostic yield of gastroscopy and colonoscopy (bidirectional endoscopy) in detecting neoplastic lesions is low. This study aimed to develop and validate a faecal immunochemical test (FIT)-based model to optimise the work-up of patients with IDA.

**Methods:**

Outpatients with IDA were enrolled in a prospective, multicentre study from April 2016 to October 2019. One FIT was performed before bidirectional endoscopy. Significant gastrointestinal lesions were recorded and a combined model developed with variables that were independently associated with significant colorectal lesions in the multivariate analysis. The model cut-off was selected to provide a sensitivity of at least 95% for colorectal cancer (CRC) detection, and its performance was compared to different FIT cut-offs. The data set was randomly split into two groups (developed and validation cohorts). An online calculator was developed for clinical application.

**Results:**

The development and validation cohorts included 373 and 160 patients, respectively. The developed model included FIT value, age, and sex. In the development and validation cohorts, a model cut-off of 0.1375 provided a negative predictive value of 98.1 and 96.7% for CRC and 90.7 and 88.3% for significant colorectal lesions, respectively. This combined model reduced the rate of missed significant colorectal lesions compared to FIT alone and could have avoided more than one-fourth of colonoscopies.

**Conclusion:**

The FIT-based combined model developed in this study may serve as a useful diagnostic tool to triage IDA patients for early endoscopic referral, resulting in considerable reduction of unnecessary colonoscopies.

## Introduction

Iron deficiency anaemia (IDA) is a major public health problem with a worldwide prevalence of 4.5 to 18% (1), accounting for up to 13% of referrals from general practitioners to gastroenterologists (2). Gastrointestinal blood loss or malabsorption are the main causes of IDA in postmenopausal women and men (2).

IDA is a predictive factor for gastrointestinal malignancies, especially in males of advanced age (3). The prevalence of gastrointestinal neoplasia in patients with IDA has been reported to be 10–20% (4). Therefore, IDA is considered an indication for urgent endoscopic referral (5). However, the primary care setting is associated with a low positive predictive value (PPV), representing only 5.8 and 1.0% for colorectal cancer (CRC) and stomach cancer, respectively (6).

After excluding celiac disease, clinical guidelines recommend diagnostic colonoscopy and gastroscopy (bidirectional endoscopy), which account for a significant workload in endoscopic units. However, no consensus has yet been reached on whether these two procedures should be carried out simultaneously, if one should take preference over the other, or if the second procedure could be omitted if the cause of IDA is detected by the first procedure (2, 7–9). Moreover, only approximately 10% of patients have significant lesions taking into consideration both endoscopic procedures (2).

Available non-invasive tests for detecting faecal occult blood loss, *Helicobacter pylori (H. pylori)* infection, or autoimmune gastritis may identify patients at risk for clinically significant gastrointestinal lesions and could help guide the work-up of patients with IDA (10, 11). The faecal immunochemical test (FIT) detects the globin from human haemoglobin (Hb) by means of monoclonal or polyclonal antibodies and allows quantification of the faecal Hb concentration. Since globin is degraded in the upper gastrointestinal tract, it cannot be detected by FIT assays. Therefore, FIT mostly detects intact globin coming from lower gastrointestinal blood loss. The 2017 Institute for Health and Care Excellence (NICE) (DG30) guidelines included FIT in the work-up of patients with lower abdominal symptoms for suspected CRC (12). Recently, FIT triage has been suggested to be useful in symptomatic patients with diagnoses other than CRC (13). However, the accuracy of FIT and its optimal cut-offs in specific subgroups of symptomatic patients are unknown. The few studies assessing the accuracy of FIT to guide the work-up of patients with IDA have been inconclusive, mainly due to their retrospective design, small sample size, heterogeneous definition of significant gastrointestinal lesions, or use of the less accurate guaiac-based faecal occult blood test (14–27).

In the current study, we hypothesised that FIT may serve as a decision-making tool in the work-up of patients with IDA, as patients with a negative test are likely to not have a significant colorectal lesion. Therefore, we aimed to build a risk prediction model based on FIT analysis to help in the endoscopic work-up of patients with IDA. The study was registered at ClinicalTrials.gov, identifier: NCT02792023.

## Methodology

### Study design, setting, and participants

This prospective multicentre cohort study was carried out in five Spanish hospitals and one Uruguayan hospital from April 2016 to October 2019. We included patients referred for bidirectional endoscopy who satisfied the following inclusion criteria: men and women aged ≥18 years with non-investigated IDA, defined as serum Hb ≤11.9 g/dL for men and ≤10.9 g/dL for women, with a serum ferritin concentration ≤30 ng/mL and transferrin saturation index (TSI) ≤16%. We also considered for inclusion patients with chronic kidney disease and/or heart failure with serum ferritin ≤200 ng/mL and TSI ≤25% and patients with an inflammatory disorder with serum ferritin ≤100 ng/mL and TSI ≤16%. These patients were diagnosed with IDA both when they consulted for symptoms of an anemic syndrome and those in whom it was detected incidentally during laboratory exams. Exclusion criteria included: hospitalised patients and those who were not candidates for endoscopic procedures due to a poor performance status; patients with any other source of blood loss, including metrorrhagia or menorrhagia; abdominal or rectal mass on physical exploration; a personal history of inflammatory bowel disease (IBD) or family history of a hereditary CRC syndrome; previous gastrointestinal surgery or having undergone colonoscopy, gastroscopy, or videocapsule endoscopy in the last 5 years; pregnancy; and refusal to participate. Moreover, none of the patients that were included in this study followed a vegan or vegetarian diet. Patients were not included for further analysis if they did not return a FIT sample, did not attend the endoscopic procedures, and/or had an incomplete gastroscopy and/or colonoscopy, unless due to neoplasia.

Colonoscopy was considered incomplete if the bowel cleansing score according to the Boston Bowel Preparation Scale (28) was <2 points at any colonic segment. The protocol was approved by the Local Ethics Committee of Hospital Universitario de Canarias, and all participants provided written informed consent.

### Outcomes

The primary outcome of this study was to develop and validate a FIT-based combined predictive model for detecting CRC and other significant colorectal lesions to prioritise patients with IDA for colonoscopy. The secondary outcomes were to compare the diagnostic accuracy of the FIT-based combined model vs. FIT at cut-offs of 2 μg Hb/g and 10 μg Hb/g in faeces to detect CRC or other significant colorectal lesions.

### Study interventions

All patients with IDA referred for bidirectional endoscopy attended an appointment with a gastroenterologist to verify inclusion/exclusion criteria and to perform a physical examination. On the day of the appointment, eligible participants received a single FIT kit (OC-Sensor^™^, EikenChemical Company, Tokyo, Japan) and were instructed to collect a sample from a spontaneous bowel movement 24 to 48 h before starting bowel cleansing for colonoscopy. Samples were returned the day of the endoscopic procedures and processed at each institution according to the manufacturer’s instructions. A blood sample was also obtained to investigate the presence of anti-transglutaminase antibodies.

Bidirectional endoscopy was scheduled within 1 month after inclusion. Both procedures were performed under conscious intravenous sedation following the protocol of each centre. Endoscopists were blind to the FIT result. At colonoscopy, biopsies were taken and therapeutic techniques applied as needed. In patients with incomplete colonoscopy for technical reasons, a colonic videocapsule endoscopy was scheduled. At gastroscopy, biopsies were taken systematically at the gastric body and antrum to rule out *H. pylori* infection and atrophic gastritis, and at the bulb and second portion of the duodenum for celiac disease diagnosis. Oral or intravenous iron therapy was initiated as needed. Patients in whom bidirectional endoscopy did not detect the cause of IDA and had an inadequate response to iron therapy were scheduled for small bowel videocapsule endoscopy.

### Definitions

The following significant colorectal lesions were considered as potential sources of IDA: CRC, advanced polyp (defined as an adenoma or serrated lesion ≥10 mm in size, and any polyp with high-grade dysplasia, tubulovillous component or traditional serrated adenoma), angiodysplasia, IBD, colitis, actinic proctitis, and solitary colorectal ulcer. In the upper gastrointestinal tract, gastric, oesophageal, and stomach cancer, angiodysplasia, gastric antral vascular ectasia, peptic ulcer, acute gastric mucosal lesions, IBD, celiac disease, gastric polyp ≥10 mm in size, Los Angeles C and D peptic esophagitis, Cameron lesions, *H. pylori*, and atrophic gastritis were considered significant lesions responsible for IDA. Significant lesions (ulcer or erosion, IBD, angiodysplasia, or neoplasia) detected by small bowel capsule endoscopy were classified as significant upper gastrointestinal lesions.

### Statistical analysis, validation, and development of the statistical model

The sample size was calculated following the four-step guide proposed by Riley et al. (29). Briefly, a confidence level of 95%, an absolute margin of error of 0.05, an outcome proportion in the study population of 0.2, a mean absolute prediction error of 0.05, eight candidate predictor parameters, an expected shrinkage factor of 0.9, and a Cox-Snell R-squared statistic anticipating a value of 0.15 were set. Based on this premise, the necessary sample size should be at least 417 individuals. In addition, our final cohort was divided by random selection into a development cohort (70%) and validation cohort (30%).

The characteristics of the two cohorts were considered as frequencies (%), means (±standard deviation [SD]), or medians with interquartile ranges (IQRs) depending on the type of variable and whether it follows a Gaussian distribution. Comparisons between the development and validation cohorts were performed using the chi-squared test, Student *t*-test, U-Mann–Whitney, or Fisher test. *p* values <0.05 were considered significant. Data were analysed using the Statistical Package for Social Sciences v. 26.0 (IBM SPSS Statistics, Armonk; NY: IBM Corp) and MedCalc v. 11.5 (MedCalc Software, Mariakerke, Belgium).

To assess the risk of presenting a significant colorectal lesion, a simple logistic regression was performed including demographic data (age, sex, and body mass index), clinical data (Charlson’s comorbidity index) (30), elapsed time from the IDA diagnosis to the date of study inclusion, treatment with antiplatelet agents, anticoagulants, non-steroidal anti-inflammatory drugs, proton pump inhibitors, and/or corticosteroids and laboratory tests (FIT [μg Hb/g in faeces], serum Hb [g/dL], serum ferritin [ng/mL], and TSI [%]). Serum parameters were measured at study inclusion, 4 weeks, and at 6 months of follow-up. Variables with *p* ≤ 0.1 were included in a multiple logistic regression analysis and expressed as odds ratios (ORs) and 95% confidence intervals (CIs). Significant variables (*p* < 0.05) and Wald’s forward variable selection method were used to construct the risk score. FIT value was entered on a logarithmic scale (Ln) to stabilise its variability. ROC curves were developed to estimate the negative predictive value (NPV), PPV, sensitivity, and specificity for FIT at cut-offs of 2 μg Hb/g, as it is the manufacturer’s defined lower detection limit, and 10 μg Hb/g in faeces according to NICE guideline criteria for symptomatic patients at low risk for CRC (11). Colonoscopy is the gold standard technique for detecting CRC, with a reported sensitivity of 94.7% (95% CI 90.4 to 92.7%) (31). Thus, ROC curves were developed to select the threshold for the positivity of the FIT-based combined model, which could prevent or delay colonoscopies without losing diagnostic value by fulfilling the following criteria: (1) sensitivity of at least 95% and (2) NPV of 98% for CRC detection. Thus, according to this threshold patients were divided into high vs. low risk for having a significant colorectal lesion or CRC. To validate the predictive value of the resultant model, we used the selected cut-off of the development model to calculate its performance by ROC curves in the validation cohort. The study accomplished the STROBE checklist for observational studies.

## Results

**Figure 1:**
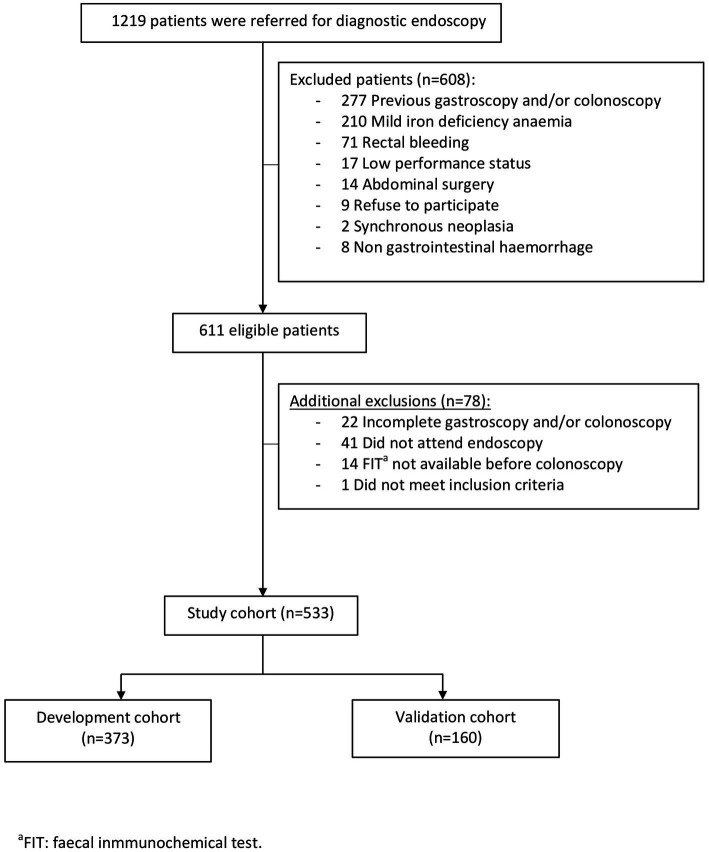
Study flow chart.

Of 1,219 consecutive patients with an endoscopic referral for IDA investigation, 611 satisfied the inclusion and exclusion criteria. Among them, 78 patients were excluded after inclusion, mostly due to not attending endoscopic procedures. Overall, 533 patients were included in the study and followed-up during 6 months. They were split up into the development (*n* = 373) and validation (*n* = 160) cohorts (Figure 1).

No significant differences were found between the development and validation cohorts regarding demographics, comorbidities, time to diagnosis, medications, or laboratory findings (Table 1). The mean age was 69.7 ± 12.8 years and females predominated over males. The mean serum Hb and MCV levels were 9.5 ± 1.4 g/dL and 77.2 ± 9.1 fL, respectively. The median serum ferritin level and TSI were 10 ng/mL (IQR 7–15.5 ng/mL) and 5.9% (IQR 4.1–8.6%), respectively. Up to 50% of patients received antiaggregants and/or anticoagulants, and almost 62% of patients were on proton pump inhibitors.

**Table 1:** Baseline characteristics of patients at the time of diagnosis of iron deficiency anaemia.

	Total (*N* = 533)	Development cohort (*N* = 373)	Validation cohort (*N* = 160)	*p*
Clinical data				
Age (years), mean ± SD	69.7 ± 12.8	69.9 ± 12.7	69.2 ± 13.1	0.560
Female, *n* (%)	340 (63.8)	236 (63.3)	104 (65.0)	0.768
Charlson’s score, mean ± SD	3.6 ± 2.2	3.6 ± 2.2	3.7 ± 2.4	0.458
Time to diagnosis (months), median (IQR)	9 (3–23)	9 (3–24)	8 (2–21)	0.168
BMI (kg/m^2^), mean ± SD	28.8 ± 5.1	28.8 ± 5.2	28.9 ± 5.0	0.773
Basal laboratory findings				
Hb (g/dL), mean ± SD	9.5 ± 1.4	9.6 ± 1.4	9.4 ± 1.6	0.265
MCV (fL), mean ± SD	77.2 ± 9.1	77.5 ± 9.4	76.6 ± 8.2	0.274
Ferritin (ng/mL), median (IQR)	10 (7–15.5)	10 (7–15)	10 (7–16)	0.991
TSI (%), median (IQR)	5.9 (4.1–8.6)	5.9 (4.1–8.4)	5.7 (4.0–9.0)	0.799
FIT (μg Hb/g of faeces), median (IQR)	6 (0–72.5)	6 (0–69.8)	5.5 (0–78.2)	0.780
Medication, *n* (%)				
Antiaggregant	204 (38.3)	139 (37.3)	65 (40.6)	0.497
NSAIDs	59 (11.1)	42 (11.3)	17 (10.6)	0.881
Oral anticoagulant	64 (11.8)	40 (10.7)	23 (14.4)	0.243
PPI	330 (61.9)	231 (61.9)	99 (61.9)	0.990

SD, standard deviation; IQR, interquartil range; BMI, body mass index; MCV, median corpuscular volume; TSI, transferrin saturation index; FIT, fecal immunochemical test; NSAID, non-steroideal anti-inflammatory drug; PPI, proton pump inhibitor. There were only missing data in BMI in 120 patients and TSI in 20 patients.

Table 2 shows the lesions found at colonoscopy and gastroscopy. There were no significant differences between the development and validation cohorts regarding endoscopic findings. Most patients (66.6%) had a normal colonoscopy. CRC was found in 68 (12.8%) patients, equally distributed between the development (*n* = 48, 12.9%) and validation cohorts (*n* = 20, 12.5%). Conversely, in the whole cohort, an upper significant lesion was detected in 62.9% at gastroscopy, with a non-neoplastic lesion being the most relevant finding. *H. pylori* infection accounted for up to 28.3% of the upper significant lesions. A gastric neoplasia was found in 17 (3.2%) patients. Concomitant lesions in the upper and lower gastrointestinal tract were found in 66 (17.7%) and 30 (18.8%) of patients in the development and validation cohorts, respectively. However, none of these patients had a synchronous neoplasia in both locations (Supplementary Table S1). Small bowel videocapsule endoscopy was performed in 75 patients, 40 (53.3%) of whom had a significant upper gastrointestinal lesion, with angiodysplasia (21%) being the most prevalent finding (Supplementary Table S2).

**Table 2:** Results of bidirectional endoscopy at the development and validation cohorts.

Findings^†^	Total (*n* = 533)	Development cohort (*n* = 373)	Validation cohort (*n* = 160)	*p*
Colonoscopy, *n* (%)				
No lesions	353 (66.6)	250 (67.0)	105 (65.6)	0.764
CRC^‡^	68 (12.8)	48 (12.9)	20 (12.5)	0.907
Advanced adenoma^‡,§^	57 (10.7)	38 (10.2)	19 (11.9)	0.545
Angiodysplasia	47 (8.8)	33 (8.8)	14 (8.8)	0.971
IBD^‡^	2 (0.4)	2 (0.5)	–	0.877
Other lesions	6 (1.1)	4 (1.1)	2 (1.3)	0.859
Gastroscopy, *n* (%)				
No lesions	198 (37.1)	137 (36.7)	61 (38.1)	0.770
*Helicobacter pylori* ^‡^	151 (28.3)	105 (28.2)	46 (28.7)	0.917
Atrophic gastritis^‡^	92 (17.3)	68 (18.2)	24 (15.0)	0.385
Peptic ulcer	41 (7.7)	27 (7.2)	14 (8.8)	0.595
Polyp ≥10 mm	25 (4.7)	18 (4.8)	7 (4.4)	0.998
Angiodysplasia	23 (4.3)	18 (4.8)	5 (3.1)	0.488
Stomach neoplasia^‡¶^	17 (3.2)	11 (2.9)	6 (3.8)	0.600
Esophagitis C/D and/or hiatal hernia with bleeding stigmata	16 (3.0)	11 (2.9)	5 (3.1)	0.913
Celiac disease^‡^	5 (0.9)	3 (0.8)	2 (1.3)	0.639

^†^The number of significant lower and upper gastrointestinal lesions may overcome the total number of patients in each cohort because there are patients with several lesions. CRC, colorectal cancer; IBD, inflammatory bowel disease. ^‡^Histological confirmation. ^§^defined as: size ≥10 mm. Villous histology and/or high grade displasia or in situ adenocarcinoma; ^¶^(gastric cancer + gastrointestinal stromal tumour).

### Fit-based combined model

Briefly, the model was constructed with the variables that were independently associated with the detection of significant colorectal lesions in the univariate analysis (Supplementary Table S3): age, sex, and FIT value. An imputation method was not applied due to absecense of missing data in these variables. Accordingly, the combined model $\hat{p}=1/(1+\exp\left(-\eta \left(x\right)\right)$ ) was built, with *p* representing the probability of suffering from a significant colorectal lesion and η(x) representing the linear predictor given by the following equation: *η*(*x*) = −3.277 + 0.473 * Ln (FIT + 1) − 0.596 * (if Sex = Female) − 0.023 * Age. The cut-off of *p* > 0.1375 was selected according to ROC curves, as it was the first cut-off that yielded at least a 95% sensitivity (95.8%) for CRC detection; its specificity, NPV, and PPV were 32.31, 98.1, and 17.3%, respectively (Table 3). The corresponding sensitivity, specificity, NPV, and PPV for detecting a significant colorectal lesion were 91.87, 38.8, 90.7, and 42.5%, respectively. The sensitivity and NPV of the FIT-based combined model at the selected cut-off were higher than those achieved at FIT cut-offs of 2 and 10 μg Hb/g in faeces, respectively (Table 4).

**Table 3:** Accuracy of FIT-based combined model cut-offs for colorectal cancer detection in the development cohort.

FIT cut-off	Sensitivity % (95% CI)	Specificity % (95% CI)	NPV % (95% CI)	PPV % (95% CI)
>0.1369	95.83 (85.7–99.5)	31.38 (26.4–36.7)	98.1 (93.2–99.8)	17.1 (12.8–22.1)
>0.137	95.83 (85.7–99.5)	31.69 (26.7–37.1)	98.1 (93.3–99.8)	17.2 (12.8–22.2)
>0.1372	95.83 (85.7–99.5)	32.00 (27.0–37.4)	98.1 (93.4–99.8)	17.2 (12.9–22.3)
>0.1375 †	95.83 (85.7–99.5)	32.31 (27.3–37.7)	98.1 (93.4–99.8)	17.3 (12.9–22.4)
>0.1395	93.75 (82.8–98.7)	32.31 (27.3–37.7)	97.2 (92.1–99.4)	17.0 (12.7–22.1)
>0.1396	93.75 (82.8–98.7)	32.62 (27.5–38.0)	97.2 (92.2–99.4)	17.0 (12.7–22.1)
>0.1408	93.75 (82.8–98.7)	32.92 (27.8–38.3)	97.3 (92.2–99.4)	17.1 (12.8–22.2)

FIT, faecal immunochemical test; NPV, negative predictive value; PPV, positive predictive value. †Selected cut-off with the highest accuracy for detecting colorectal cancer.

**Table 4:** FIT cut-offs accuracy comparison to detect colorectal cancer or any significant colorectal lesion in the development and validation cohorts.

	Colorectal cancer			Significant colorectal lesion		
	FIT 2 μg/g	FIT 10 μg/g	Combined model 0.1375	FIT 2 μg/g	FIT 10 μg/g	Combined model 0.1375
Development cohort						
Sensitivity (%)	91.7	81.2	95.8	86.2	72.4	91.9
Specificity (%)	44.6	61.2	32.3	52.8	69.6	38.8
NPV (%)	97.3	95.7	98.1	88.6	83.7	90.7
PPV (%)	19.6	23.6	17.3	47.3	53.9	42.5
Validation cohort						
Sensitivity (%)	90.0	90.0	90.0	85.4	78.2	87.3
Specificity (%)	46.3	62.1	41.4	56.2	73.3	50.5
NPV (%)	97.0	97.8	96.7	88.1	86.5	88.3
PPV (%)	19.4	25.4	18	50.5	65.5	48

FIT, faecal immunchemical test; CRC, colorectal cancer; SCL, significant colorectal lesion; NPV, negative predictive value; PPV, positive predictive value.

**Figure 2:**
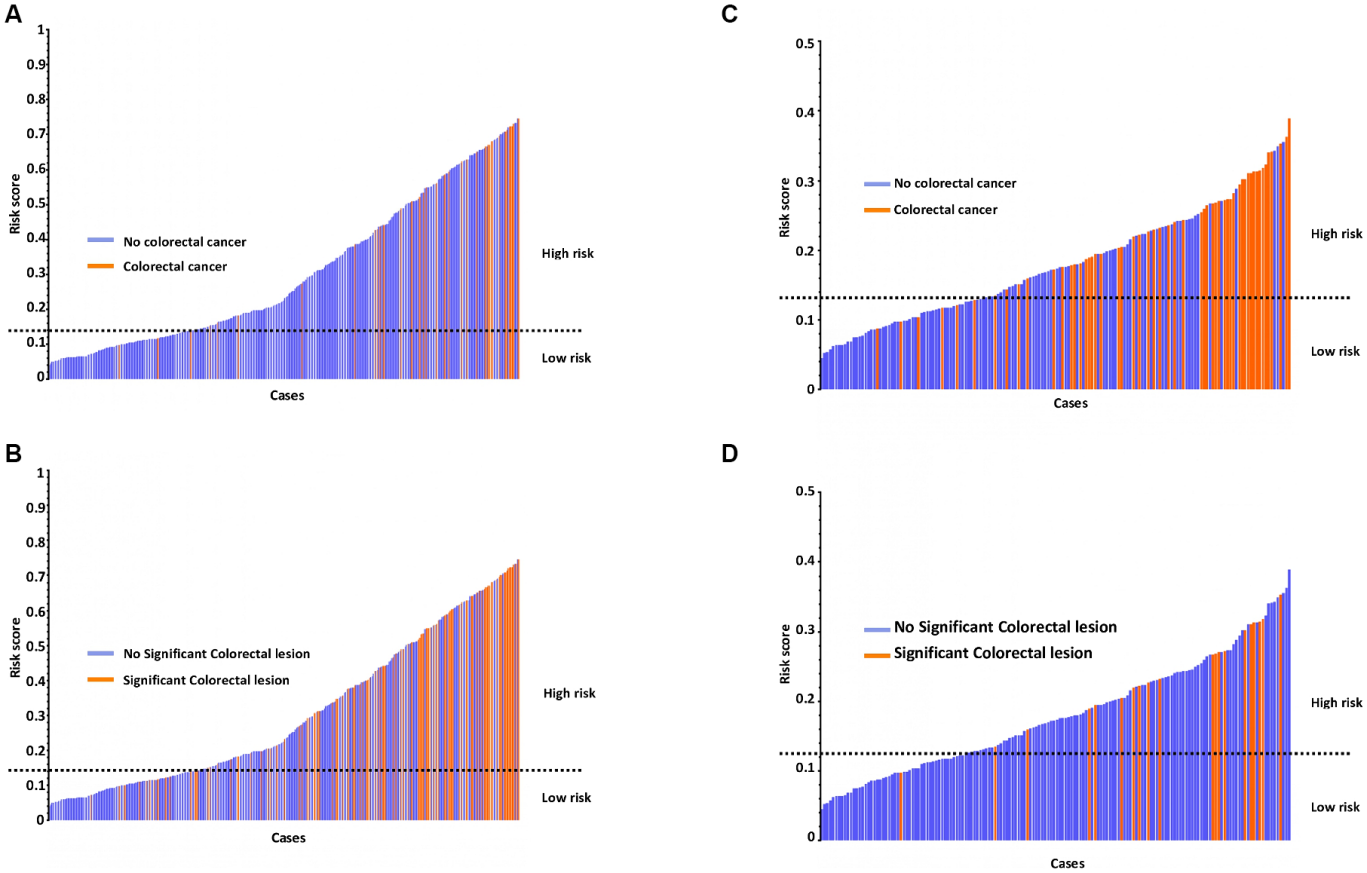
Risk score for the detection of CRC or any significant colonic lesion according to the FIT-based combined model in the development cohort **(A,B)** and validation cohort **(C,D)**.

According to the FIT-based combined model, 107 colonoscopies (28.7%) would have been prevented or delayed. Consequently, the development cohort was divided according to the FIT-based combined cut-off into groups of patients at high risk (>0.1375) and low risk (≤0.1375) for having a significant colorectal lesion or specifically CRC. As shown in Figures 2A,B, up to 10 significant colorectal lesions had not been identified, even when using the FIT-based combined model, though only two of them were CRC. However, the number of significant colorectal lesions and CRC that would be missed with this model is considerably lower than the number of missed lesions found when the FIT is applied alone, either at 2 or 10 μg Hb/g in faeces (Supplementary Table S4). In addition, there were no significant differences in the demographic and clinicopathological features when CRC patients were classified as high vs. low risk (Supplementary Table S5).

### Study validation

A total of 160 patients were included in the validation cohort. The corresponding sensitivity, specificity, NPV, and PPV for detecting significant colorectal lesions or only CRC using the FIT cut-offs of 2 and 10 μg Hb/g faeces are shown in Table 4. Applying the FIT-based combined model developed in the validation cohort at the selected cut-off of 0.1375, the sensitivity and NPV for detecting a significant colorectal lesion were 87.27 and 88.3%, respectively. The corresponding sensitivity and NPV increased for CRC detection, up to 90.0 and 96.7%, respectively (Table 4). Based on this threshold, the validation cohort was divided into high (>0.1375) and low risk (≤0.1375) groups regarding significant colorectal lesions or CRC (Figures 2C,D). Using this model, 60 colonoscopies (37.5%) would be prevented or delayed and 10 significant colorectal lesions, 2 of them CRC, would be missed (Supplementary Table S4). No significant differences were found in the demographic and clinicopathological features of CRC patients classified as high vs. low risk (Supplementary Table S5).

### Proposed work-up algorithm in patients with IDA

**Figure 3:**
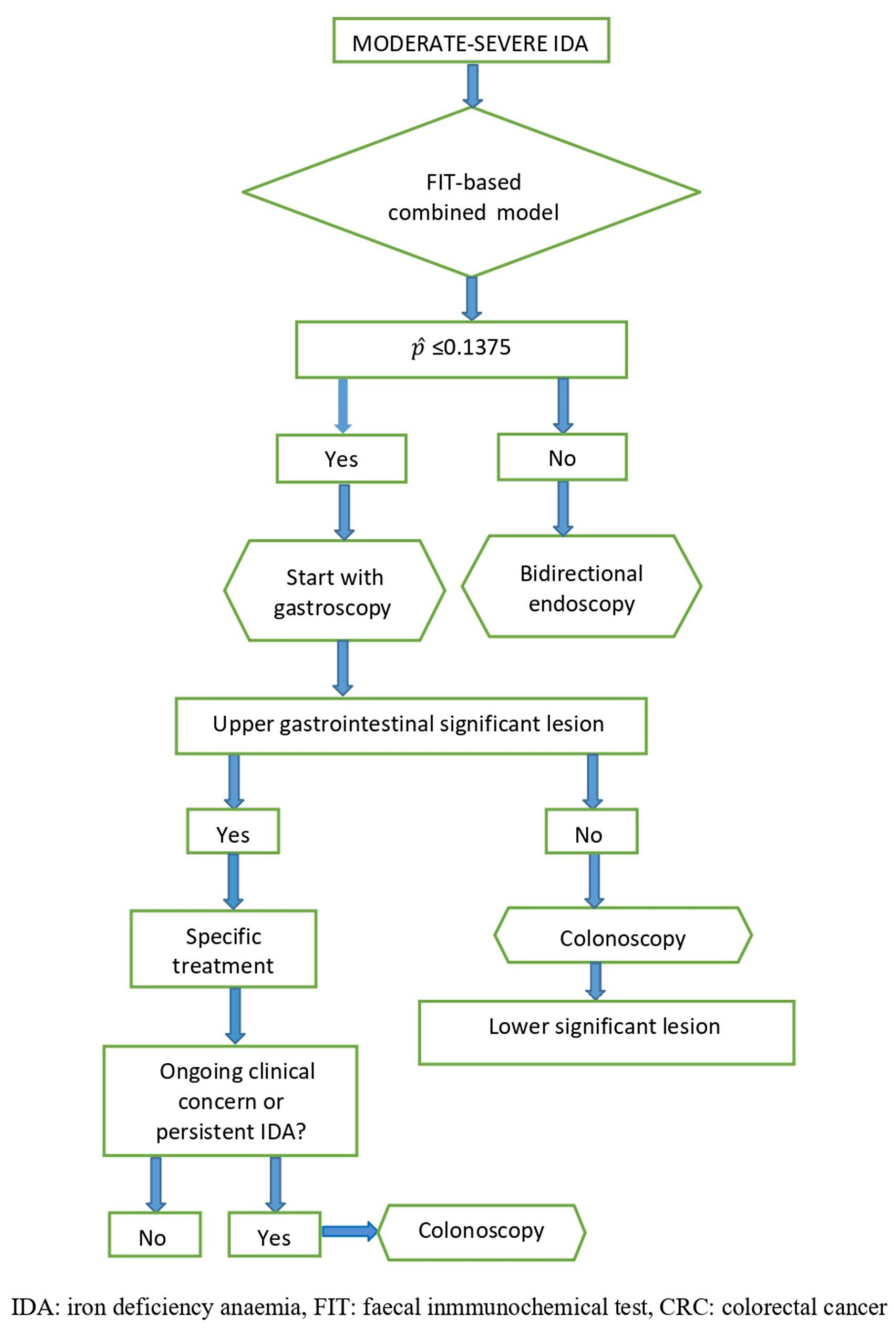
Proposed algorithm for the work-up of patients with moderate-severe IDA based on the FIT-based combined model.

Considering the higher sensitivity and NPV of the proposed FIT-based combined model, the low rate of concomitant lesions at the upper and lower gastrointestinal tract, and the absence of synchronous neoplasia in both locations, we propose the following algorithm for the work-up of patients with IDA (Figure 3): in patients with a FIT-based combined model score ≤ 0.1375, gastroscopy should be performed first, as the probability of having a significant colorectal lesion is very low. If a significant upper gastrointestinal lesion is detected in this procedure, it should be treated as needed. After that, a safe netting for the patient must be ensured by assessing whether the IDA persists, if there are any additional symptoms, or if clinical concern remains to undergo further colorectal investigation. On the other hand, if the FIT-based combined model score is >0.1375, bidirectional endoscopy is recommended. Supplementary Figures S1, S2 show how this algorithm was applied for the development and validation cohorts, respectively.

### Online calculator

**Figure 4:**
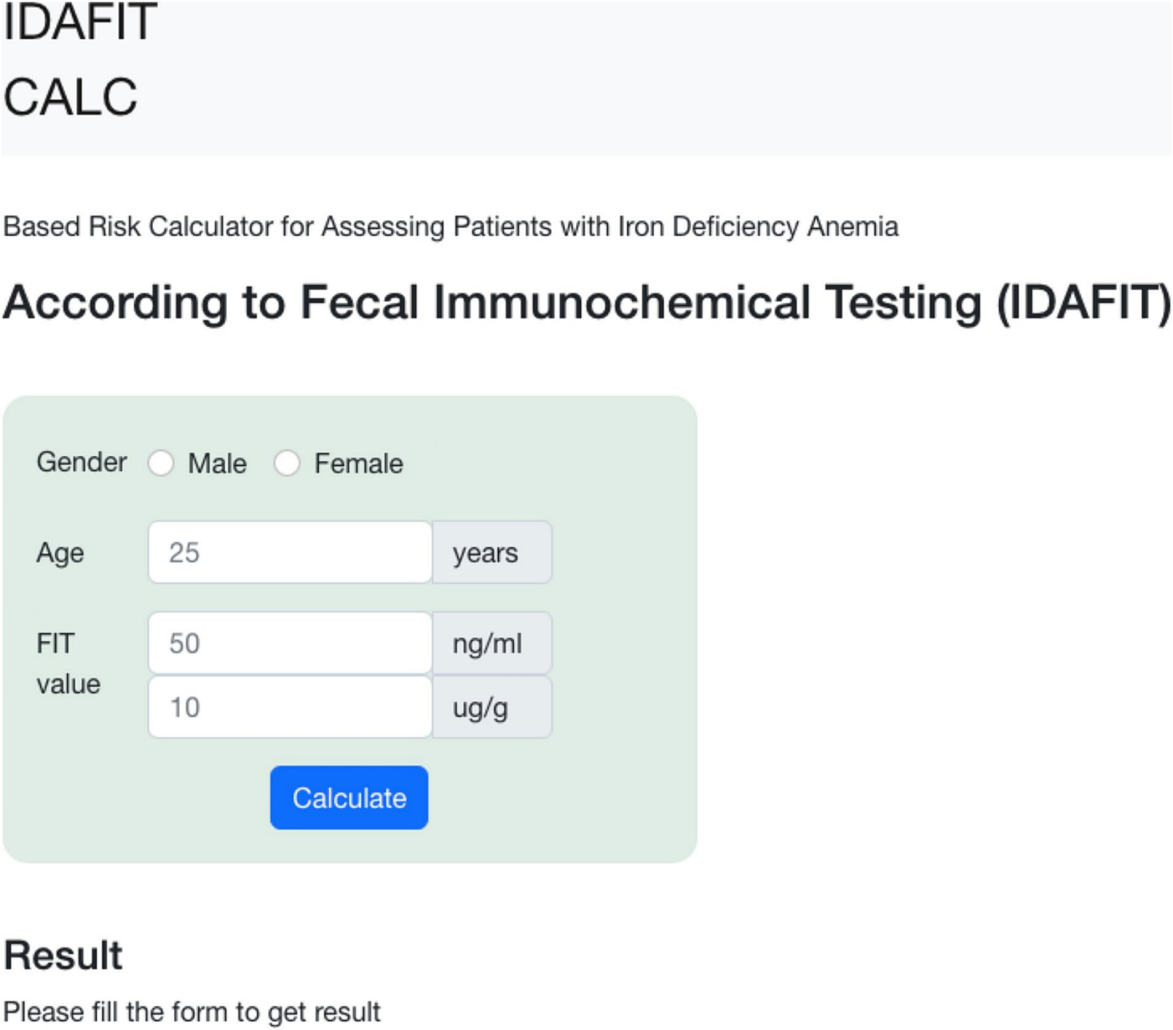
Online mobile application-based risk calculator to guide patients with iron deficiency anaemia according to faecal immunochemical testing (IDAFIT score).

The formula derived from the FIT-based combined model allows the development of an easy-to-use clinical online calculator by entering only three variables (age, sex, and the FIT value). This approach provides the likelihood of having a significant colorectal lesion and/or CRC in patients with IDA. This calculator is freely available online at https://idafit.org/ (Figure 4) and could help guide the work-up of patients with IDA, avoiding unnecessary explorations.

## Discussion

The current study suggests that a FIT-based combined model including age, gender, and FIT value could be of great help in the work-up of patients with IDA, avoiding bidirectional endoscopy in about one-third of patients. Interestingly, the high NPV of the model allows CRC to be ruled out with high confidence, preventing a substantial number of unnecessary colonoscopies.

This study has several strengths. First, to the best of our knowledge, this is the first time that a prospective study has specifically assessed a FIT-based combined model in the work-up of patients with IDA. Second, its multicentre nature, strict inclusion criteria involving only patients with mild-severe non-investigated IDA who completed a high-quality bidirectional endoscopy, a follow-up of at least 6 months, and confirmation of results in a validation cohort reinforce the power of the study. Third, the analysis incorporates a rationale threshold for detecting significant colorectal lesions that resulted from the FIT-based combined model, which improved the diagnostic accuracy of the FIT cut-offs (2 and 10 μg Hb/g faeces) previously recommended for symptomatic patients. Fourth, an online calculator constructed with the variables included in the FIT-based combined model (age, gender, and FIT value) facilitates the decision-making process for initiating the work-up of IDA patients with either bidirectional endoscopy or gastroscopy alone.

We acknowledge that there are some limitations to overcome. First, the low PPV of the developed model for detecting a significant colorectal lesion means that a substantial volume of patients would be misclassified as high risk, resulting in unnecessary referrals for colonoscopy. However, the high NPV observed in this study is more confident than the PPV in ruling out significant colorectal lesions. As a tool for triaging patients, this model could avoid or delay approximately one-third of colonoscopies, with a rate of 5.8% CRC missed in the whole cohort, which is considerably lower than the previously reported 9.12% when a FIT cut-off of 10 μg Hb/g faeces is applied in symptomatic patients (31). Second, the exclusion of 77 (12.6%) patients that fulfilled the inclusion criteria but did not attend endoscopy or had incomplete explorations could introduce a selection bias, although this small percentage of patients is unlikely to alter the results of the study. Third, we did not perform an external validation, which was not possible because of the advent of the Covid-19 pandemic. Nevertheless, the internal validation could mitigate the extent of overfitting in the developed cohort.

IDA is a major health issue that leads to an unaffordable burden of endoscopic procedures in endoscopic units, which has recently worsened due to the COVID-19 pandemic. A recent meta-analysis of 12 retrospective studies suggested that FIT could be useful for prioritising symptomatic patients for colonoscopy (32). However, methodological flaws in most of these studies make this statement questionable. First, several studies included patients with IDA together with others having vague symptoms (abdominal pain, change in bowel habits), in whom the likelihood of having a significant colorectal lesion is very low. Second, this meta-analysis showed that FIT had a low sensitivity (64%) for detecting significant colorectal lesions in patients with IDA. Third, some studies assessed the efficacy of FIT together with the stool guaiac test, which is already considered obsolete for clinical practice. Fourth, among the studies that specifically assessed the diagnostic yield of FIT, only two of them had a prospective design, and both had heterogeneous definitions for IDA (22, 24). In addition, the FIT threshold was not reported in one study (22) and was >10 μg Hb/g in faeces in another (24), though it is the recommended cut-off for symptomatic patients at low risk of having CRC according to NICE guidelines (12).

On the other hand, the NICE guidelines do not include FIT for the assessment of patients with high-risk symptoms for CRC, including those with IDA (12). D’Souza et al. (31), Chapman et al. (33), and Lanas et al. (34), recently evaluated the diagnostic accuracy of FIT in these patients and concluded that FIT could be useful for ruling out CRC in this setting. Interestingly, they suggested that the threshold for FIT positivity should be reduced at the lower limit of detectability provided by the manufacturer (0–2 μg Hb/g faeces for OC-Sensor^™^) to have a high sensitivity for CRC. The results of the current study are in line with this finding and reinforce the concept that a lower cut-off is needed to rule out CRC with greater confidence. We found that a FIT threshold ≥10 μg Hb/g of faeces was not accurate enough to rule out significant colorectal lesions, including CRC, due to its low sensitivity (81–90%). This limitation was overcome by the FIT-based combined model developed in our study, which allowed us to select a rational threshold with a sensitivity of 95.8% and NPV 98.1% for CRC detection. Moreover, the sensitivity and NPV were also higher than 90% for detecting a significant colorectal lesion. In any case, the choice of the threshold should be a trade-off among the number of CRC cases and significant colorectal cancer lesions missed and the number of patients referred for colonoscopy considering the potential complications that could be associated with the endoscopic procedures and the waiting list dealt with by endoscopic units.

An interesting finding of our study was that most significant lesions causing IDA were found in the upper gastrointestinal tract, with *H. pylori* infection being the most prevalent cause, a condition that can be diagnosed with high accuracy by non-invasive tools (35). *H. pylori* eradication therapy solves IDA in most of these patients. Further studies assessing the role *H. pylori* eradication prior to endoscopic referral are needed to evaluate the number of gastroscopies that could be avoided in this setting.

Currently, bidirectional endoscopy is recommended for all patients with IDA (9, 36). This approach is reinforced by the lack of methods capable of discriminating between upper or lower gastrointestinal lesions. The current study suggests that the implementation of a model developed by combining the patient’s age, gender, and FIT value substantially improves the diagnostic accuracy for detecting CRC and other significant colorectal lesions. Interestingly, the resultant model threshold of 0.1375 allowed us to categorise patients as high risk (score > 0.1375) or low risk (score ≤ 0.1375) of having significant colorectal lesions. Based on this model, an algorithm can be proposed for the work-up of patients with IDA (Figure 3).

In summary, the current FIT-based combined model increases the diagnostic accuracy of significant colorectal lesions and provides a sensitivity equivalent to colonoscopy for detecting CRC in patients with IDA. This model provides a diagnostic tool by which to triage these patients for urgent endoscopic referral, preventing a substantial number of unnecessary colonoscopies.

## Ethics statement

The studies involving humans were approved by Comité de Ética de la Investigación con Medicamentos (CEIm), Complejo Hospitalario Universitario de Canarias. The studies were conducted in accordance with the local legislation and institutional requirements. The participants provided their written informed consent to participate in this study.

## Author contributions

GH: Conceptualization, Data curation, Investigation, Methodology, Project administration, Resources, Supervision, Validation, Visualization, Writing – original draft, Writing – review & editing. EQ: Conceptualization, Data curation, Funding acquisition, Investigation, Resources, Supervision, Validation, Visualization, Writing – original draft, Writing – review & editing. DM-A: Investigation, Project administration, Validation, Visualization, Writing – review & editing. GR: Investigation, Resources, Validation, Visualization, Writing – review & editing. GM: Investigation, Resources, Validation, Visualization, Writing – review & editing. NF: Investigation, Resources, Visualization, Writing – review & editing. LC: Investigation, Resources, Validation, Visualization, Writing – review & editing. M-VÁ-S: Investigation, Resources, Validation, Visualization, Writing – review & editing. CO: Investigation, Resources, Validation, Visualization, Writing – review & editing. DR-A: Investigation, Resources, Validation, Visualization, Writing – review & editing. CG: Investigation, Resources, Validation, Visualization, Writing – review & editing. IA: Investigation, Resources, Validation, Visualization, Writing – review & editing. DN-P: Investigation, Methodology, Writing – review & editing. MC-P: Investigation, Resources, Validation, Visualization, Writing – review & editing. EG-D: Data curation, Formal analysis, Software, Writing – review & editing. AG-G: Funding acquisition, Investigation, Methodology, Resources, Visualization, Writing – review & editing.
