# Supplementary material for: Development and validation of a faecal immunochemical test-based model in the work-up of patients with iron deficiency anaemia
Source: Front Med (Lausanne). 2024 Jun 25;11:1407812. doi: 10.3389/fmed.2024.1407812 (PMC11231424; doi:10.3389/fmed.2024.1407812)
Supplement: Supplementary file 1 [file Data_Sheet_1.PDF]

**Supplementary Table 1. Patients with concomitant upper and lower gastrointestinal lesions in the development and validation cohorts.**

| Detected lesions | Development Cohort (n= 66) | Validation Cohort (n= 30) |
|---|---|---|
| **Colonoscopy findings, n (%)** | | |
| CRC | 25 (37.9) | 8 (26.7) |
| Advanced adenoma †‡ | 20 (30.3) | 11 (36.7) |
| Angiodysplasia | 20 (30.3) | 9 (30.0) |
| IBD | 1 (1.5) | - |
| Other lesions | 1 (1.5) | 2 (6.7) |
| **Gastroscopy findings, n (%)** | | |
| *Helicobacter pylori* infection | 33 (50.0) | 20 (66.7) |
| Atrophic gastritis | 17 (25.8) | 6 (20.0) |
| Peptic ulcer | 7 (10.6) | 3 (10.0) |
| Polyp ≥ 10 mm | 3 (4.5) | 1 (3.3) |
| Angiodysplasia | 7 (10.5) | 2 (6.7) |
| Stomach neoplasia § | 1 (1.5) | - |
| Esophagitis C/D or hiatal hernia with stigmata of bleeding | 4 (6.1) | 1 (3.3) |
| Celiac disease | - | - |

FIT: faecal immunochemical test· CRC: colorectal cancer,
†Histological confirmation. ‡Size ≥ 10 mm, villous histology, and/or high-grade dysplasia or in situ adenocarcinoma.
IBD: inflammatory bowel disease
§Gastric cancer + gastrointestinal stromal tumour, no patients had concomitant neoplastic lesions in both locations.

**Supplementary Table 2.** Findings in patients who underwent small bowel videocapsule endoscopy

| Findings | Total N= 75 | Development cohort N=56 | Validation cohort N=19 | *P* |
|---|---|---|---|---|
| No lesion, n (%) | 35 (46.7) | 26 (46.4) | 9 (47.4) | 0.943 |
| Angiodysplasia, n (%) | 21 (28.0) | 17 (30.4) | 4 (21.1) | 0.560 |
| Erosions, n (%) | 11 (14.7) | 7 (12.5) | 4 (21.1) | 0.455 |
| Inflammatory bowel disease, n (%) | 3 (4.0) | 3 (5.4) | - | 0.567 |
| Bleeding, n (%) | 1 (1.3) | 1 (1.8) | - | 0.999 |

**Supplementary Table 3.** Univariate analysis of variables associated with the detection of significant colorectal lesions in the development and validation cohorts.

| | Development Cohort | | | | Validation Cohort | | | |
|---|---|---|---|---|---|---|---|---|
| | **Detected (N = 123)** | **Not detected (N = 250)** | **OR (CI 95%)** | ***P*** | **Detected (N = 55)** | **Not detected (N = 105)** | **OR (CI 95%)** | ***P*** |
| **Clinical features** | | | | | | | | |
| Age (years) , mean ± SD | 73.6 ±10.7 | 68.1 ± 13.3 | 1.04 (1.02-1.06) | <0.001 | 72.0 ± 11.7 | 67.8 ± 13.7 | 1.03 (1.00 - 1.06) | 0.045 |
| Female, n (%) | 66 (53.7) | 170 (68.0) | 0.54 (0.35-0.85) | 0.009 | 28 (50.9) | 76 (72.4) | 0.40 (0.20 - 0.78) | 0.009 |
| Charlson´s score, mean ± SD | 4.0 ± 1.9 | 3.3 ± 2.3 | 1.16 (1.05-1.29) | 0.002 | 4.1 ± 2.1 | 3.5 ± 2.5 | 1.11 (0.97 - 1.28) | 0.120 |
| Time to diagnosis (months), median (IQR) | 8 (2-23.0) | 10 (3-24) | 0.91 (0.77-1.07) | 0.276 | 9 (2- 19.5) | 6 (3 - 21.5) | 0.95 (0.74 - 1.21) | 0.609 |
| BMI (kg/m$^2$), mean ± SD | 28.6 ± 4.7 | 28.9 ± 5.5 | 0.99 (0.94 - 1.04) | 0.635 | 29.3 ± 4.1 | 28.7 ± 5.4 | 1.02 (0.95 - 1.10) | 0.587 |
| **Basal laboratory findings** | | | | | | | | |
| Hb (g/dL), mean ± SD | 9.41 ± 1.56 | 9.69 ± 1.30 | 0.87 (0.74 - 1.01) | 0.088 | 9.51 ± 1.41 | 9.40 ±1.77 | 1.04 (0.85 - 1.27) | 0.712 |
| MCV (fL), mean ± SD | 76.6 ± 9.32 | 78.0 ± 9.48 | 0.98 (0.96 - 1.01) | 0.169 | 76.8 ± 8.55 | 76.5 ±8.15 | 1.00 (0.97 - 1.05) | 0.837 |
| Serum ferritin (ng/mL), median (IQR) | 10 (7-15) | 10 (7-15.3) | 1.07 (0.80-1.43) | 0.498 | 10 (8-16) | 10 (6-15.5) | 1.16 (0.74-1.81) | 0.802 |

| | | | | | | | | |
|---|---|---|---|---|---|---|---|---|
| TSI (%) , median (IQR) | 5.6 (3.8-8.0) | 6.0 (4.3-8.7) | 0.77 (0.51-1.17) | 0.166 | 6.1 (3.3 - 8.0) | 5.5 (4.2 - 9.6) | 0.74 (0.44 - 1.27) | 0.334 |
| FIT (µg/g faeces) Median (IQR) | 63.8 (7.4-222.2) | 1.8 (0-18.1) | 1.65 (1.46-1.86) | <0.001 | 66.6 (15.0 - 210.2) | 0.6(0 - 15.0) | 1.69 (1.41 - 2.02) | <0.001 |
| **Medication, n (%)** | | | | | | | | |
| Antiaggregant | 40 (32.5) | 99 (39.6) | 0.73 (0.47 - 1.16) | 0.211 | 23 (41.8) | 42 (40.0) | 1.08 (0.56 - 2.09) | 0.866 |
| NSAIDs | 8 (6.5) | 34 (13.6) | 0.44 (0.20 - 0.99) | 0.046 | 4 (7.3) | 13 (12.4) | 0.55 (0.17 - 1.79) | 0.422 |
| Anticoagulant | 12 (9.8) | 28 (11.2) | 0.86 (0.42 - 1.75) | 0.725 | 7 (12.7) | 16 (15.2) | 0.81 (0.31 - 2.11) | 0.814 |
| PPI | 80 (65.0) | 151 (60.4) | 1.22 (0.78 - 1.91) | 0.428 | 34 (61.8) | 65 (61.9) | 1.00 (0.51 - 1.95) | 0.991 |

OR: odds ratio, CI: confidence interval, SD: standard deviation, IQR: interquartile range, BMI: body mass index, Hb: haemoglobin, MCV: median corpuscular volume, TSI: transferrin saturation index, FIT: faecal immunochemical test, NSAID: non-steroidal anti-inflammatory drug, PPI: proton pump inhibitor.

**Supplementary Table 4.** Number of significant colorectal lesions and colorectal cancer that would be missed using an FIT value of 2 or 10 µg Hb/g in faeces compared to the FIT-based combined model.

| | Development Cohort | | Validation Cohort | |
|---|---|---|---|---|
| | CRC | SCL | CRC | SCL |
| FIT 2 µg Hb/g faeces | 2 | 8 | 4 | 17 |
| FIT 10 µg Hb/g faeces | 2 | 12 | 9 | 34 |
| FIT-based combined model threshold (0.1375) | 2 | 7 | 2 | 10 |

FIT: faecal immunochemical test, CRC: colorectal cancer, SCL: significant colorectal lesion

**Supplementary Table 5.** Comparison of demographic and clinicopathological features of patients with colorectal cancer classified as high vs. low risk according to the FIT-based combined model cut-off.

| | **Development cohort N= 48** | | | **Validation cohort N = 20** | | |
|---|---|---|---|---|---|---|
| | **Low risk (N = 2)** | **High risk (N = 46)** | ***P*** | **Low risk (N = 2)** | **High risk (N = 18)** | ***P*** |
| Age, years, mean± SD | 75.5 ± 6.4 | 74.4 ± 9.2 | 0.868 | 79.0 ± 11.3 | 72.6 ± 10.3 | 0.417 |
| Female, n (%) | 2 (100) | 21 (45.7) | 0.224 | 2 (100) | 10 (55.6) | 0.495 |
| Location[†], n (%) | | | | | | |
| - Proximal | 2 (100) | 31 (67.4) | 0.743 | 2 (100) | 11 (61.1) | 0.857 |
| - Distal colon/rectum | -- | 13 (32.6) | -- | -- | 7 (38.9) | -- |
| FIT, µg Hb/g faeces, | | | | | | |
| Median (IQR) | -- | 200.0 (61.7; 492.9) | 0.002 | 0 (0; 0) | 135.5 (75; 435.1) | 0.011 |

SD: standard deviation, IQR: interquartile range, FIT: faecal immunochemical test; FIT-based combined model threshold was 0.1375.

[†]Relative to the splenic flexure.
