# Supplementary material for: Development and validation of a faecal immunochemical test-based model in the work-up of patients with iron deficiency anaemia
Source: Front Med (Lausanne). 2024 Jun 25;11:1407812. doi: 10.3389/fmed.2024.1407812 (PMC11231424; doi:10.3389/fmed.2024.1407812)
Supplement: Supplementary file 2 [file Data_Sheet_2.pdf]

Supplementary Figure 1

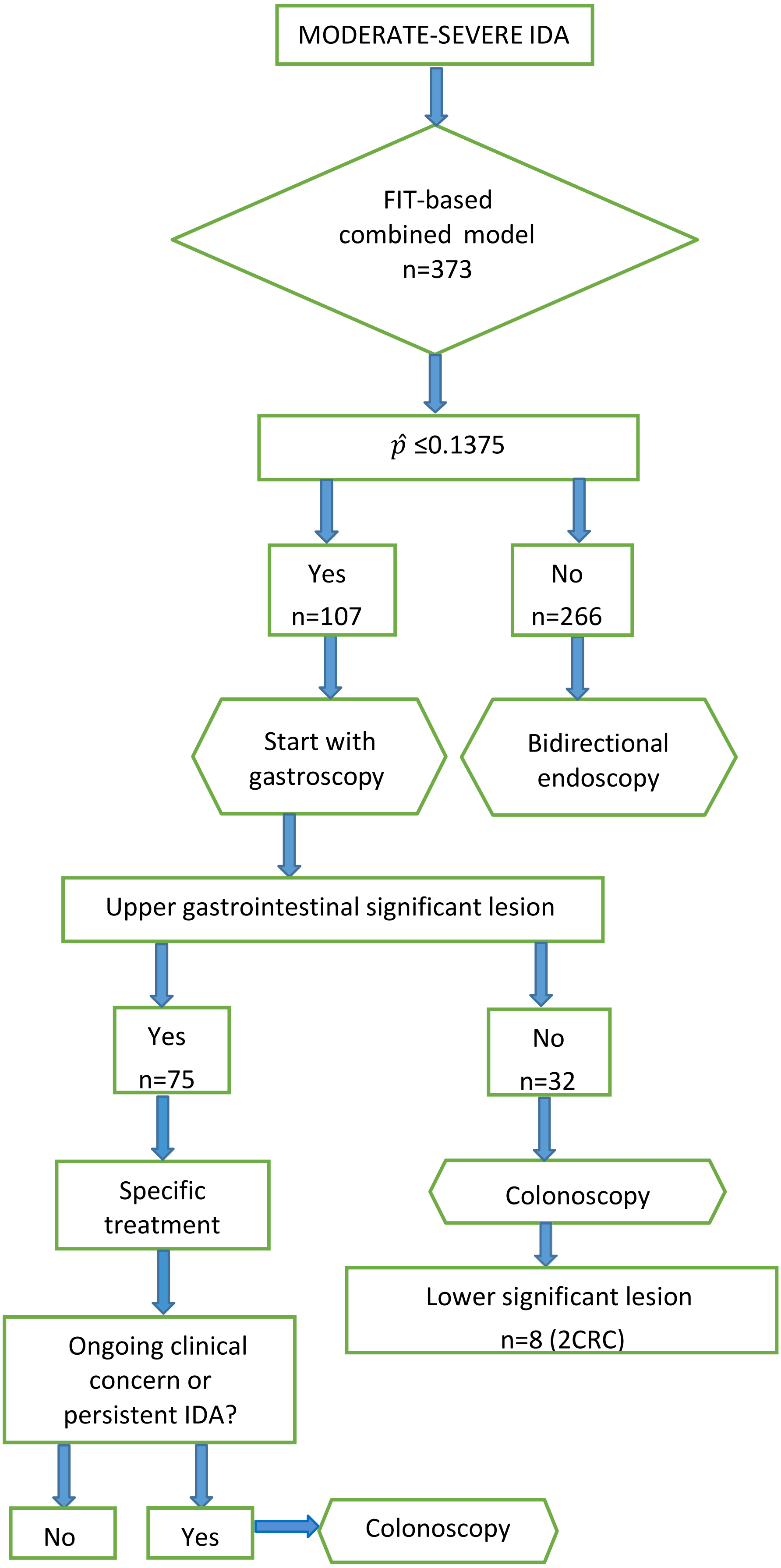

IDA: iron deficiency anaemia, FIT: faecal inmmunochemical test, CRC: colorectal cancer

Supplementary Figure 2

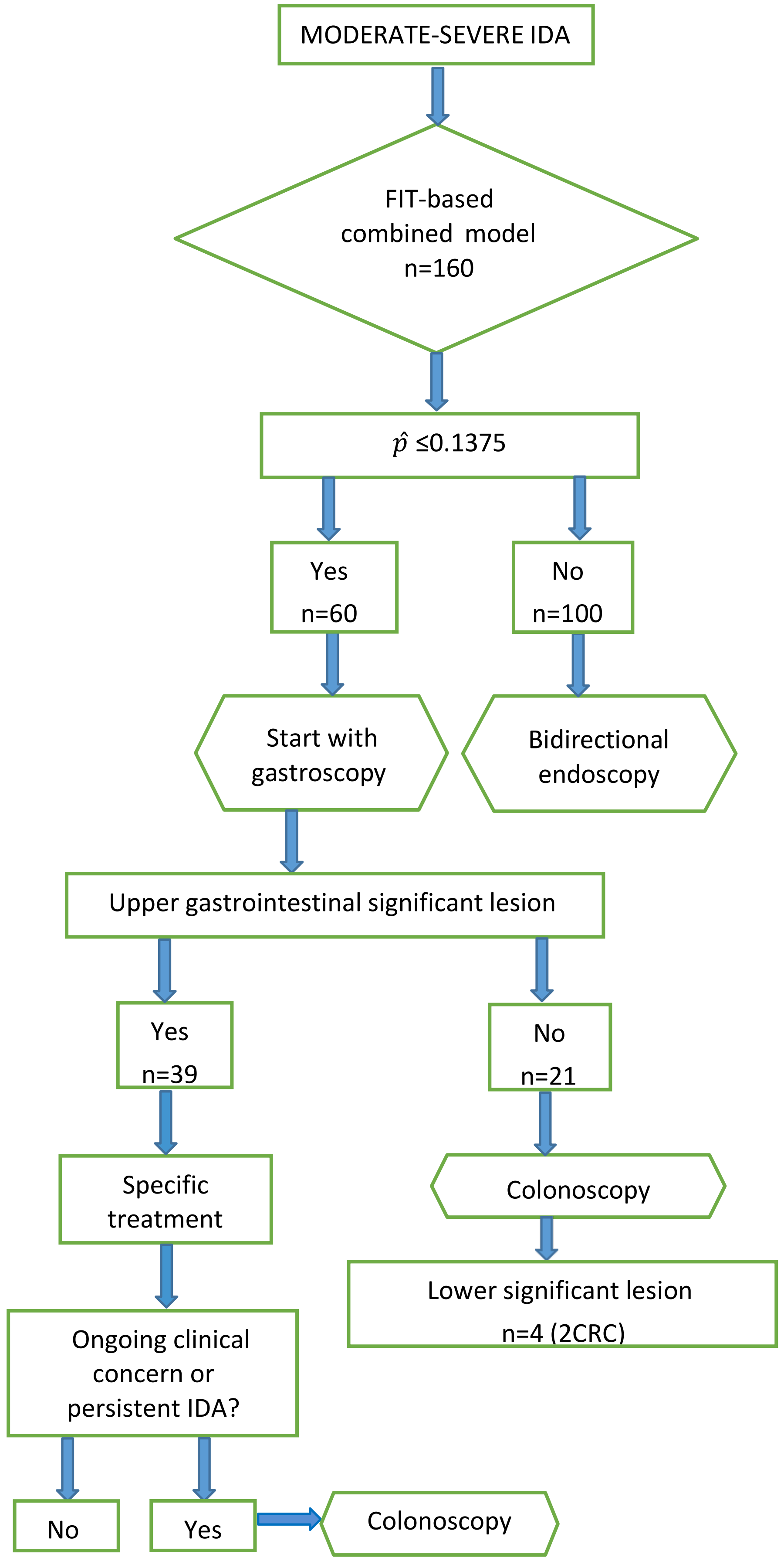

IDA: iron deficiency anaemia, FIT: faecal inmmunochemical test, CRC: colorectal cancer
